# Obstacles to routine immunization in Madagascar: Structural, relational and cultural constraints

**DOI:** 10.1016/j.jvacx.2023.100348

**Published:** 2023-07-13

**Authors:** Henintsoa Joyce Valentina Ramaroson, Chiarella Mattern, Elise Huysmans, Hobisandratra Razafiarimanana, Emilia Brazy-Nancy, Mendrika Haritiana Ranaivoharimina, Dolorès Pourette

**Affiliations:** a Health & Social Sciences, Epidemiology and Clinical Research Unit, Pasteur Institute of Madagascar (IPM), BP 1274 Ambatofotsikely Avaradoha, 101 Antananarivo, Madagascar; b Applied Human Sciences, Arts & Sciences Faculty, Montreal University, Montreal, Canada; c Prospective Anthropology Laboratory (LAAP), Catholic University of Louvain, Louvain-La-Neuve, Belgium; d Catholic Relief Services (CRS), Antananarivo, Madagascar; e French National Research Institute for Sustainable Development – Ceped (IRD, Paris University, INSERM) – Paris, France

**Keywords:** Immunization, Anthropology, Madagascar, Epidemic, Perceptions

## Abstract

This article proposes an overview of anthropological studies concerning childhood immunization in Madagascar, where vaccine coverage still remains low. The COVID-19 epidemic has heightened the reticence of populations to be vaccinated in the public health centres of this country. It appears necessary to study the factors which may impact immunization practices and its representations. The principal results of these studies describe immunization from the perspective of parents of children under five years of age. The role that they attribute to a vaccine is essentially that of reinforcing the health of children against illness. There is a misunderstanding of combination vaccines, one vaccine generally being associated with one disease. The findings also show a complex relationship with immunization, injections and the health passport. Finally, uptake of immunization or not is motivated by the structural and local context such as the perceived frailness of the newborn baby, the practice of post-partum confinement, or the lack of knowledge about the vaccine. Lack of understanding of immunization and mistrust of it are signs of a fragile health system. An in-depth analysis of immunization and the impact of COVID-19 on its perception will provide a clearer view of the fragility of the immunization programmes implemented in Madagascar.

## Highlights

• Parents have little knowledge of the role of immunization on vaccine-preventable diseases.
• There is confusion about immunizations, their frequency and number, which can lead to reluctance by parents.
• The immunization record, which facilitates access to care, is the main reason why parents use childhood vaccines.
• Immunization is not a priority, its delay or interruption is not considered serious for the child's health.

## 1 Introduction

The contribution of the social sciences to the prevention and combat of infectious diseases is increasing, in particular via the analysis of the social parameters related to this menace [1]. The major part of these contributions concerns the use and supply of medicines, the circulation of counterfeit medicines on the markets of the Southern hemisphere [2], [3], [4], [5], [6] and immunization [7], [8], [9]. A decline in vaccine coverage on a global scale is currently observed, as much in countries of the North as in the Southern hemisphere [10].

Qualitative studies on immunization in the African continent show that behaviour with respect to immunization varies from one country to another and the origin of this is multifactorial: concern about vaccine safety, lack of information about vaccines, greater reticence towards new vaccines not included in the initial vaccine programme such as that against HPV [11] or the influenza vaccine [12]. Added to these factors is the influence of friends, neighbours or family opposed to immunization [7], religious denominations, an example of which is a study in Katanga (immunization, being biomedical, is against Congolese traditions and prohibitions) [16], vaccines being supposedly free but which nevertheless incur expenses [17], [19], and lastly the geographical distance from immunization centres [20].

In Madagascar, the PEV (Extended Immunization Programme) was started in 1975. It covers 10 life-threatening diseases for the under-fives (tuberculosis, poliomyelitis, tetanus, diphtheria, whooping cough, measles, haemophilus influenzae, hepatitis B, Rota virus and Pneumococcus). Immunization activities are coordinated by the DPEV. Each activity follows a 5-level circuit: (1) the DPEV is responsible for the national coordination of each program implemented, (2) regional coordination is provided by the regional health authority, (3) activities are then communicated to the health districts, (4) the CSB (representing the commune) convenes the AC (community agents) to inform them, and finally, (5) the AC are responsible for sharing the information in their respective area. At the present time vaccine coverage for the routine vaccines is low: 37.8% [13]. Routine childhood immunization infantile is confronted with numerous challenges and limits. A study conducted in Antananarivo in 2019 revealed that vaccine coverage is influenced by the high mobility of individuals and the heterogeneity of the environments, added to which is the lack of motivation of community workers [14]. According to this same study, populations receiving fewer immunization interventions or refusing vaccines were principally those from religious movements, working in the informal sector and living in underprivileged areas or yet again, migrant populations (*ibid*). A study conducted in 3 rural areas of Madagascar (Analanjirofo, Anosy, Atsimo Andrefana) in 2015 shows that the long distance from immunization centres and lack of information and communication are the principal reasons for non-immunization [15]. More specifically the absence of the child from their community on the immunization day was the principal reason for non-immunization in this three same areas [16]. Moreover, the low involvement of community agents in immunization activities, is an important factor in the outcome of immunization activities. The explanation is that the remunerations which they receive for immunization and awareness-raising activities do not provide the necessary motivation [17]. Concerning information and communication, the mothers consider the radio and healthcare services to be the main source of information [15]. These various challenges in Madagascar have been reinforced recently by the 2019 measles epidemic [18] followed by the COVID-19 pandemic.

The objective of this article is to review the individual or collective reasons leading to low vaccine coverage in Madagascar via a description of knowledge and perceptions of routine vaccine practices in children under 5 years of age. These data presented here are from three qualitative studies conducted between 2013 and 2019. There are still numerous barriers to routine childhood immunization in Madagascar.

## 2 Materials and methods: Three studies on maternal and childhood health and immunization in Madagascar

This article presents the results from three qualitative studies conducted between 2016 and 2019: two studies conducted by the SaSS (Health & Social Sciences) team of the Epidemiology and Clinical Research Unit of IPM (Madagascar Pasteur Institute) [15], [29] and a Master's thesis [21]. These anthropological studies describe practices and perceptions of childhood care from birth to five years of age, including social representations and childhood vaccine practices. These 3 studies took place in 5 different geographical areas of the island: Antsohihy (North), Manakara (South-east), Moramanga rural (East), Moramanga rural (East), and Morondava (West).

### 2.1 Edras: Study of the factors determining health care uptake in pregnant women and children aged under 5 years (2018)

This study, conducted by the SaSS team, combined quantitative and qualitative methodologies to document the determinants of health care uptake by pregnant women and children. The quantitative study mobilised the disciplines of clinical epidemiology, health economics and the geographic information system. The qualitative study was conducted with socio-anthropological tools, i.e., semi-structured interviews. Data collection was articulated around 5 themes: use of preventive and curative care during pregnancy, use of healthcare establishments, the birth process and choice of place of delivery, use of care during the neonatal period, use of a method of family planning before and after the pregnancy by the women and use of preventive and curative care for children under 5 years of age, including immunization. Three districts were included: Antsohihy, Moramanga and Manakara. These areas are supported by USAID funding via the programmes MSH Mikolo and Mahefa Miaraka.

The qualitative surveys were conducted in 2018 at Antsohihy and Manakara. In addition, a retrospective analysis of qualitative data collected in 2013 at Moramanga was conducted. The qualitative section consisted of a total of 202 semi-directive interviews with 28 healthcare workers (community workers, midwife, doctor, *matrone*), 111 women (mothers and pregnant women), 49 men and 14 grandmothers.

### 2.2 NeoVac: Neonatal immunization against hepatitis B in sub-Saharan Africa (2016–2019) – A feasibility study

The programme NeoVac (Neonatal Immunization against hepatitis B in sub-Saharan Africa) aimed to assess the possibility of developing an appropriate strategy for vaccinating newborn infants against hepatitis B in the first 24 h of life. Neovac was conducted, in three countries, including Madagascar, and has in 2 phases: (1) an anthropological study to document the feasibility of hepatitis B vaccination at birth before setting up a vaccine programme, and (2) a randomised controlled trial in the community proposing immunization of newborns within the first 24 h, to assess the impact of the intervention on the coverage of anti-hepatitis B vaccine at birth and on neonatal mortality.This study was conducted by the SaSS team in collaboration with the IRD (*Institut de Recherche pour le Developpement*) and the Paris Pasteur Institute.

In the case of Madagascar, a 4-month immersion in a Moramanga village (Ampasipotsy gare) provided a reconstruction of the therapeutic itineraries of 16 pregnant women, from pregnancy to childbirth. This continuous monitoring of pregnant women punctuated by a first interview with the individual (n = 16) before the observation of her delivery, then a second after the birth (n = 15). A last interview was conducted when the newborn infant was between two to four weeks old (n = 13). In addition, interviews (n = 39) and focus groups (n = 5) were conducted with caregivers involved in mother and child care (elderly women, parents, *matrones*, tradipractitioners, personnel qualified in childbirth and/or immunization, and community workers).

### 2.3 Master's thesis: Uptake and refusal of immunization in mothers of children under 5 years old (2016)

This 2016 study was for the master's thesis of H.J.V Ramaroson, directed by D. Pourette. It was anthropological research on how immunization is perceived by mothers of children aged under 5 years.

The findings of this research are based on semi-structured interviews conducted with 11 mothers of children aged under 5 years, living in the Namahora *fokontany* (traditional Malagasy village), in the urban area of Morondava. This zone was selected due to the low vaccine coverage in the region, 23% in the period 2012–2013 [22]. The themes discussed were the notion of prevention in childcare, curative treatments for children (medical or traditional) and the acceptance or not, of immunization.

A similar method of analysis was used for all 3 studies, the interviews conducted during these 3 surveys were recorded and a Word file of the full transcript was made and then translated from Malagasy to French. They were then codified using a list of codes determined before data collection, a code being a label used to mark a concept (e.g., care pathway). For each project, analytical grids were designed and completed.

## 3 Results

The results describe the social representations of immunization and care for children under 5 years, the main obstacles to immunization in the study areas, and leverage points. It should be noted that despite the varied geographic zones studied, the results of the three studies are in agreement, nevertheless any differences will be described.

### 3.1 The mothers' knowledge about immunization

The first result concerns the role of the mother in childhood immunization. Globally, in all 5 zones, children's health is women's business. Therefore, the mother is responsible for complying with the immunization schedule, taking the child for immunization. It is also the mother who knows where the health passport (*carnet de santé*) is kept, if it is not lost. The interviews also describe the role of the other women of the family (especially grandmothers) in ensuring continuity of child care, if the mother is temporarily not available. The mothers know more about immunization than the fathers. This may be explained by the more frequent exposure of the mothers to messages and awareness-raising about immunization.

In all zones the results show that the mothers have little knowledge about immunization. More specifically the nature of vaccines is not well understood: VPD (vaccine-preventable diseases), single dose and multidose vaccines, mass and routine immunizations. Great confusion was revealed by the parents' narratives in all zones.

Firstly, there was limited understanding on the subject of VPD, the vaccines administered during childhood, the signs, methods of transmission, the existence of treatments. Although some diseases (measles, polio) are known due to awareness-raising campaigns or because some mothers have experienced them, little information is provided about them. In the NeoVac study, the parents were able to name the vaccines, but the latter were rarely associated with specific diseases. Thus, the absence of certain symptoms or VPD in the local compass of disease understanding, such as hepatitis B for example, renders the vaccine optional for the population, unnecessary and of secondary importance.

Next, there is confusion among mothers between mass and routine immunizations: why repeat, during mass immunization sessions, vaccines already administered in the routine immunization schedule? Vaccines administered during immunization campaigns appear useless for parents because they are repeated. These vaccines described by the mothers as reinforcing the routine vaccines, leads the parents to question the efficacy of the first vaccines at birth. This representation of mass immunizations fuels the belief that campaigns are politically motivated, reinforcing rumours about vaccines: they could make the child sterile; the vaccines are out of date and may produce long-term sequelae. The persistence of these rumours leads to the mistrust of community workers and other persons administrating the vaccines, in charge of awareness-raising and administration during mass campaigns. The legitimacy of community workers in health matters is therefore challenged in the case of immunization. In few cases, mothers are favourable to mass immunizations when they have experienced specific diseases (measles, polio): for example, they may have known people who have died from these diseases, or they have had the disease themselves [21].

Lastly, the studies also show incomprehension about combined vaccines, the vaccine associated with a single disease is much better understood. There is little relationship established between vaccines and the diseases covered, except in rare cases where the children of parents have had the experience of a VPD. Vaccine uptake is in fact enhanced if the parents have an understanding of diseases, their symptoms of their sequelae. Participants who were in favour of vaccines because of fear of the disease also consider that the injections reduce the child's frailness, frailness acquired at birth [21].

### 3.2 What the use of the health passport reveals about the relationship with immunization

The principal role attributed by the parents to immunization is a general reinforcement of *hery fiarovana* (immune system). In the three studies, the majority of mothers define immunization as the best way of preserving the child's health: the children vaccinated may become ill but will recover more easily than those who are not vaccinated.

A wide variability in vaccine status between siblings was noted, some children had completed their immunization schedule, others not. This variability in behaviour is even more difficult to analyse that the health passports (booklets indicating the vaccine status of the child) are often in poor condition or lost and renewed, without recording the vaccines given before the loss of the booklets. The health passport, given by the health professional to the parents at the time of the child's first immunization, is theoretically used to record the vaccines administered to the child and the date of the next immunization appointments. It also contains useful information for the mothers such as advice on nutrition and breast-feeding, or action to take in case of symptoms. The last pages of the health passport are exclusively used for the immunization schedule. Lastly, this booklet also gives access to treatment and consultations in health centres when the child is ill, it is thus a consultation booklet and a record of the child's health.

“We need the vaccine passport is the child ever has to go to hospital. If the child is not fully vaccinated, the doctor will refuse to treat them and will send them away. I think that happens up to the age of 6 years. Some parents lie and say they have lost the passport. The parents risk being told off by the doctor otherwise. That happens mainly with children under 6 I think. Even if the child is seriously ill.” (Woman, 30 years, shopkeeper, Morondava) [21].

In addition to access to care for the child, the presentation of a “fully completed” health passport ensures a better reception from the healthcare personnel according to parents. To have a better relationship with the healthcare personnel is another incentive to encourage parents to have their children vaccinated. The Edras study showed that the quality of this relationship depends on the behaviour of the mother on the day of immunization, it is a question of being a “good mother” that is, “be clean, have beautiful clothes (or ideally, new) for herself or her child and to have the resources necessary in case of shortages to nevertheless be able to get the immunization” [23].

In the 5 zones, the women complained that if the passport is of lost or damaged, the healthcare personnel scold them and can refuse access to consultations or to immunization sessions. The qualitative survey, Edras, reveals failure to adhere to the immunization schedule and thus the intervals between vaccines. Adherence to the vaccine schedule is not a priority for parents in the care of their children. Moreover, the first vaccine is deliberately deferred by the mothers, considering the child to be still “fragile” during the first two months after birth. In other circumstances, because the immunization of the child is a task usually attributed to the mother, the delay in the immunization schedule will be justified by her timetable. For example, the appointments may clash with other activities considered more important than immunization, such as *mifana* (confinement*)* described below, or the daily chores. The predominance given to meeting the requirements of other activities to the detriment of immunization, indicates that immunization at birth is not a priority for the newborn infant. Immunizations are then delayed or not completed.

### 3.3 Obstacles to immunization

In general, total refusal of immunization was not apparent from these studies. There was rather a delay or refusal of some vaccines caused by structural, contextual or geographic obstacles.

#### 3.3.1 Structural obstacles

The Edras and NeoVac studies revealed structural difficulties with immunization which here are described according to 4 axes: availability of human resources in the health services for the administration of vaccines, availability and accessibility of vaccines and problems linked to the open vial policy.

Concerning the availability of human resources, the three studies showed that healthcare personnel were frequently unavailable in the CSB ('basic' health centres). In the absence of healthcare personnel, no substitute is designated to ensure continuity of service in the CSB. Mothers' narratives indicate that walking for several days to reach the nearest centre is not uncommon.

Moreover, in the three projects, parents often remark vaccine shortages on the days scheduled for the immunization. According to Edras project, immunization has to be paid for in case of shortage. Therefore, the mothers, not having prepared the necessary sum beforehand, leave and return when the stock is replenished. Lastly, what the healthcare personnel call the “open vial policy” is a significant structural obstacle to immunization access: multidose vials for tuberculosis (BCG), measles, mumps, rubella and chickenpox (VAR) contain a number of doses (10 to 20) which must be used within a specific period. When the number of children who have come for one of these two vaccines is insufficient (at least 5), the immunization is cancelled. Again, very numerous narratives of the mothers in these 5 zones regret the absence of vaccine administration due to this “open vial policy” and despite their attendance at the centre.

#### 3.3.2 *Mifana* and shame: A socio-cultural environment hostile to compliance with the immunization schedule

Besides reasons related to the structural context, the findings also reveal local contexts which can impede immunization: the practice of *mifana* or confinement after childbirth, the perceived vulnerability of the young infant at birth, the mothers being made to feel inferior by the healthcare personnel in the health centres due to their clothing and physical appearance.

At birth, the newborn infant is perceived as too frail to be vaccinated. An infant at birth is considered “fragile”. For this reason, the newborn will not be able to stand the pain of the injection or the effects of the injected vaccine. The baby therefore remains confined with their mother during the period commonly known as *mifana* (the practice of confinement widespread in the zones studied) ranging from a few days to a few months. During *mifana*, the mother and her newborn remain confined and must avoid going outside which is perceived as “hostile” and would increase the child's fragility. This latter is considered more exposed to death and illness coming from outside, and must therefore remain with their mother in the home to avoid all risk.

“I have been in ”mifana“ up to now. But today I had to go outside to have the baby vaccinated. Even if you are in ”mifana“, when you have to have the baby vaccinated, you must go! For the rest of the time I continue to rest at home, I do not do much exercise yet”, Woman 23 years, Moramanga [19].

This practice aims to conserve their health. We find the same idea of fragility in the NeoVac study, where vaccine refusal is explained by fragility of the newborn. Moreover, the immunization may be deferred if the child falls ill, because the parents consider the child too fragile to receive a vaccine, in this case they fear possible side effects.

In addition to these factors, the Edras study reveals the notion of shame felt by the mothers. This shame is associated with a feeling of being humiliated by the healthcare personnel due to their clothes and physical appearance described as “slovenly”. This may be an obstacle to attending the health centres, thus adversely affecting routine immunizations. The Edras study describes the importance for the mothers and their newborn babies to be “well dressed” and “presentable” and to have a well-kept health passport with an up-to-date immunization schedule.

“The hospital is not for people who lack equipment. And I am frightened of the hospital. It is simpler to have the baby at home… You have to take a lot of things to there… It's a question of food. You have to eat good meals there. When you do the confinement [at home], breadfruit is enough for us… Because even if we are in a rural environment, the doctors, the medical personnel don’t come from a rural environment but the people on top… Also, we have to have clean clothes, otherwise the midwife will be cross with us. Or otherwise, say here at the hospital, to be in good health, you must be clean. But us, we don't have time to do that…” Mother, Manakara.

This extract illustrates the idea that the quality of reception will depend on the ability of the mothers to conform to an image expected by the CSB. Therefore, the mothers justify delayed immunization by this feeling of shame, the avoidance being to escape the shame and stigmatisation which occurs in some relationship dynamics between caregivers and those cared for.

#### 3.3.3 Geographic obstacles

The NeoVac and Edras studies show that geographic remoteness is secondary in the choice to not vaccinate the child: the distance between the home and the CSB and the isolation of some zones. These justifications however appear first in the mother's narratives, legitimising the delay or refusal to vaccinate. However, closer examination of the narratives of seeking care for children and the analysis of the available care options in a limited therapeutic area (NeoVac) indicates that when they feel the necessity, distance is no longer an obstacle. An example is the narrative of a mother interviewed in Moramanga rural concerning her choice to give birth in a health centre, the therapeutic path of whom is retraced in detail. This mother lives several kilometres from a health centre and several metres from a well reputed *matrone* in this *fokontany*. However, she consciously chose to give birth in a health centre of the same quality, but further away because of close relationship with the caregiver in charge.

Moreover, the findings of the studies also report the fact that delivery in a health centre does not guarantee systematic immunization of the child at birth, although in this case the geographic distance would be zero. This is explained by the perceived vulnerability of the child (considered too fragile at birth as described above) but also by vaccine shortages or again the “open vial policy” described above.

## 4 Discussion

The studies reviewed show that vaccine uptake and compliance with the schedule depend upon a series of interconnected factors: the relationship between the child's parents and the healthcare personnel, the practice of traditional care at birth, understanding and perceptions of immunization and vaccine-preventable diseases, and also the role attributed to vaccines. In common with numerous studies on immunization, these anthropological surveys conducted in Madagascar revealed late uptake of vaccines or hesitancy to certain categories of vaccines, rather than complete refusal of immunization [24].

The data also indicate that the populations have a poor understanding of immunization, reducing its usefulness to that of a reinforcement of the immune system. This confusion can lead to doubts concerning the efficacy of some of the vaccines: if the child falls ill after being vaccinated, the parents tend to feel that the immunization has failed or is useless. To this is added the problem of the social non-existence of certain vaccine-preventable pathologies. This decreases the perception of vulnerability to these diseases whereas the feeling of vulnerability to disease, as in the case of influenza H1N1, strongly enhances vaccine uptake [25].

The lack of knowledge of these diseases, hepatitis B for example, coupled with a general mistrust of the public health system [9] can fuel reticence to new vaccines. The literature which evokes this lack of knowledge concerns mainly the new vaccines, for which awareness raising campaigns are needed, in particular for those against hepatitis B (*ibid*.) or papillomavirus [26].

Under other circumstances, the lack of motivation towards immunization may also be combined with the fact that the VPD are perceived as being without danger or serious complications. At the same time, hesitancy and worry concerning immunization can also be explained by the increasing numbers of recommended or compulsory vaccines in the immunization schedule [27].

These studies also reveal an instrumentalization of immunization: obtaining a health passport through immunization is, for the parents, one of the main motivations in vaccine uptake, providing access to healthcare for the child and enhancing the caregiver-cared for relationship. Any incentives to immunization remain delicate, in particular because they are conditioned by social constraints, the parents' knowledge of immunization and the relationship between healthcare personnel and the parents. As a general rule, the narrative of the participants in these studies did not evoke any true opposition to immunization, in contrast to other research where the safety of vaccines and their conflict with religious beliefs were the main reasons for refusal. The parents' attitude corresponded to what Sobo et al. [28] called “selective vaccinators”, that is, the fact of taking into account the child's environment or specific diseases, in the decision to vaccinate or not.

The relationship of the populations with the health structures and healthcare personnel appears to be an essential factor in the parents' attitude to vaccines: the need for access to care in the CSB, the desire to have a good relationship with the healthcare personnel, the medical opinion concerning mass immunizations. The confidence and the relationship with the doctor are crucial factors when new mothers make a decision concerning the immunization of their children [29]. An unsatisfactory reception from health workers, added to concerns of cost and access to immunization services add to an individual's preoccupations about immunization [30]. On the other hand, a strong recommendation from doctors and tradipractitioners could strengthen the intention to seek immunization [39, 40]. Lastly, this article reveals shame as an obstacle to vaccine uptake by the mothers. This shame is fostered by the gaze of others (the health personnel or parents) [31] and can lead to the logic of exclusion for the parents, not only preventing immunization but also preventing them seeking care when the child falls ill.

The three studies in this article present meaningful information on routine immunization, on its barriers and levers, but fail to provide a sufficiently in-depth analysis of the subject. In fact, the results of these studies mainly concern the perceptions of the parents. Other aspects of immunization remain to be studied. In particular the parameters of organisation and information in addition to the relationship between the various stakeholders involved in immunization and the implementation of the current national immunization strategy.

It should be noted that the parameter of communication is not explored in the studies in immunization in Madagascar, knowing that the influence of the media and the social networks can also cause the efficacy of vaccines to be questioned [32]. In other countries, media over-exposure of accidents and health scandals undermines confidence in the public health authorities and by extension, to immunization itself [33].

We finally remark that the malfunction and organisational weaknesses of the PEV was reflected in the measles epidemic (2018–2019): decreased vaccine coverage, higher levels of refusal in the large towns. To mitigate these problems revealed during the measles epidemic, WHO conducted two studies in 2019: an anthropological study on immunization [34] and a quantitative study on lost immunization opportunities [35]. Following these studies, an anthropological study on immunization, conducted by IPM, is on-going to highlight these various aspects of childhood immunization.

## 5 Conclusion

The studies reviewed in this article show that childhood immunization is known to parents but as a means to reinforce the child's health, however its mechanism (frequency, combined vaccines, vaccine-preventable diseases, mass immunizations…) remains poorly understood by the latter. In practice, immunization is not considered by mothers a childcare priority. It can therefore be deferred or discontinued by the parents. It is primarily a means to access care in a health centre in case the child falls ill. We note that the obstacles connected to availability and access to vaccines are increasing. Lastly, immunization is revealed as a guarantee to a good relationship between the parents and the health workers in the CSB. Firstly, failure to complete the immunization enhances transmission of disease requiring the use of antimicrobials. Secondly, shame which may be felt by the mother when the health passport is incomplete or when she fails to present all the other characteristics of a good mother, can prevent her consulting the doctor if the child falls ill.

## Declaration of Competing Interest

The authors declare that they have no known competing financial interests or personal relationships that could have appeared to influence the work reported in this paper.

## Data Availability

Data will be made available on request.

## References

[1] Frid-Nielsen S.S., Rubin O., Baekkeskov E. (2019). The state of social science research on antimicrobial resistance. Soc Sci Med.

[2] Schultsz, C, de Vries D. SPECIAL SOC AMR curriculum [Internet]. Sonar-Global; 2020 [cited 2022 Jul 4]. <https://www.sonar-global.eu/special-soc-amr/>.

[3] Desclaux A. L’automédication comme pratique collective. Pratiques et fonctions dans la vente de compléments nutritionnels au Sénégal. « Automédication Choisie Ou Subie » - Rech En Géographie Médecine Générale Sociol [Internet]; 2015 [cited 2022 Jul 4]. <https://automed.hypotheses.org/cotonou2015/desclaux>.

[4] Baxerres C, Cassier M, Chabrol F, Haxaire C. Trente-cinq ans d’anthropologie du médicament en Afrique : retour sur l’étude des marchés informels, des hôpitaux et des usages pharmaceutiques. Anthropol Santé Rev Int Francoph Anthropol Santé [Internet]; 2017 May 15 [cited 2022 Jul 4];(14). <https://journals.openedition.org/anthropologiesante/2462>.

[5] Pemunta NV, Nzefa LD, Ngo V. Self-medication within the context of medical pluralism in Yaounde, Cameroon; 2019.

[6] Quet M. Impostures pharmaceutiques : médicaments illicites et luttes pour l’accès à la santé- fdi:010075080- Horizon [Internet]. Paris: La découverte; 2018 [cited 2022 Jul 4]. 248 p. <https://www.documentation.ird.fr/hor/fdi:010075080>.

[7] David YY, Yomi TF, Yao KO, Atta K, Blé YM, Drissa K. Facteurs Associés à la Réticence de la Vaccination Contre la Poliomyélite chez les Mères dans la Commune d’Abobo, Abidjan: Cas du Quartier Samaké; 2019.

[8] Dubé È., Ward J.K., Verger P., MacDonald N.E. (2021). Vaccine hesitancy, acceptance, and anti-vaccination: trends and future prospects for public health. Annu Rev Public Health.

[9] Giles-Vernick T., Traoré A., Bainilago L. (2016). Incertitude, Hepatitis B, and infant vaccination in West and Central Africa. Med Anthropol Q.

[10] Unicef. WHO and UNICEF warn of a decline in vaccinations during COVID-19 [Internet]; 2020 [cited 2022 Jan 14]. <https://www.unicef.org/press-releases/who-and-unicef-warn-decline-vaccinations-during-covid-19>.

[11] Ward J.K., Peretti-Watel P., Larson H.J., Raude J., Verger P. (2015). Vaccine-criticism on the internet: new insights based on French-speaking websites. Vaccine.

[12] Zamir C.S., Israeli A. (2017). Knowledge, attitudes and perceptions about routine childhood vaccinations among jewish Ultra-Orthodox mothers residing in communities with low vaccination coverage in the jerusalem district. Matern Child Health J.

[13] INSTAT. Enquête de Couverture Vaccinale (ECV) 2021 [Internet]; 2021 [cited 2022 Mar 24]. <https://www.instat.mg/p/enquete-de-couverture-vaccinale-ecv-2021>.

[14] Unicef. Approche atteindre chaque enfant (A.C.E. / Équité), composante urbaine; 2019.

[15] Unicef. Étude des déterminants socio-culturels pour l’adoption des pratiques familiales essentielles dans les régions d’intervention de l’Unicef, rapport sur les résultats de l’analyse des données quantitatives; 2015.

[16] Unicef. Analyse des données existantes relatives aux refus et abandons de vaccination. Profil d’analyse par région en matière de vaccination polio à Madagascar (Analanjirofo, Atsimo Andrefana, Anosy); 2016.

[17] Mattern C, Cripps A, 2016. Les Agents communautaires, une figure ambivalente, in Rapport d'activité IPM 2016 [Internet], [cited 2023 Mar 23]. <marhttps://www.pasteur.mg/wp-content/uploads/2017/06/Rapport-d-activites-IPM-2016.pdf>.

[18] Nimpa M.M., Andrianirinarison J.C., Sodjinou V.D., Douba A., Masembe Y.V., Randriatsarafara F. (2020 Mar). Measles outbreak in 2018–2019, Madagascar: epidemiology and public health implications. Pan Afr Med J.

[19] Institut Pasteur de Madagascar. Étude des déterminants du recours aux soins des femmes enceintes et des enfants de moins de 5 ans (Edras); 2019.

[20] Institut Pasteur de Madagascar. Néovac : vaccination néonatale contre l’hépatite B; 2019.

[21] Ramaroson HJV, Pourette D. Perception des vaccinations de routine et de masse par les mères. Cas du fokontany de Namahora, région Menabe. In: FEMMES, ENFANTS ET SANTÉ À MADAGASCAR Approches anthropologiques compares; 2018. p. 173–84. (Anthropologies & Médecines).

[22] Unicef. Children in Madagascar : a promise of a future. Situation analysis of the mother and child 2014 [Internet]; 2014 [cited 2022 Jun 30]. <https://www.unicef.org/madagascar/en/reports/children-madagascar-promise-future>.

[23] Huysmans E. Quand la santé questionne les liens: anthropologie de la santé maternelle et infantile en milieu rural dans le Sud-Est de Madagascar; 2019.

[24] Roberts J, Mitchell LM. « Ton corps, ta décision » : perspective anthropologique sur la réticence à la vaccination contre le VPH. In: La santé publique à une ère marquée par le doute : origines religieuses et culturelles de l’hésitation des Canadiens face à la vaccination [Internet]. Les Éditions de l’Université de Sherbrooke (ÉDUS); 2019 [cited 2021 Dec 8]. p. 262. <https://savoirs.usherbrooke.ca/handle/11143/16021>.

[25] Massé R., Weinstock D., Désy M., Moisan C. (2011). Perceptions populaires du risque et savoirs experts en contexte de pandémie: le cas du A (H1N1) au Québec. Anthropol Santé Rev Int Francoph Anthropol Sante.

[26] Santhanes D., Wong C.P., Yap Y.Y., San S.P., Chaiyakunapruk N., Khan T.M. (2018). Factors involved in human papillomavirus (HPV) vaccine hesitancy among women in the South-East Asian Region (SEAR) and Western Pacific Region (WPR): a scoping review. Hum Vaccines Immunother.

[27] Barbacariu C.L. (2014). Parents’ refusal to vaccinate their children: an increasing social phenomenon which threatens public health. Proc-Soc Behav Sci.

[28] Sobo E.J., Huhn A., Sannwald A., Thurman L. (2016). Information curation among vaccine cautious parents: Web 2.0, Pinterest thinking, and pediatric vaccination choice. Med Anthropol.

[29] Benin A.L., Wisler-Scher D.J., Colson E., Shapiro E.D., Holmboe E.S. (2006). Qualitative analysis of mothers’ decision-making about vaccines for infants: the importance of trust. Pediatrics.

[30] Muñoz D.C., Llamas L.M., Bosch-Capblanch X. (2015). Exposing concerns about vaccination in low-and middle-income countries: a systematic review. Int J Public Health.

[31] Diallo Y. Pauvreté et maladie. Une Médecine Inhospitalière Difficiles Relations Entre Soignants Soignés Dans Cinq Capitales Afr Ouest Paris APAD-Karthala; 2003.

[32] Steffens M.S., Dunn A.G., Wiley K.E., Leask J. (2019). How organisations promoting vaccination respond to misinformation on social media: a qualitative investigation. BMC Public Health.

[33] Thiongane O. Le vaccin à marche forcée. Vie Idées [Internet]. 2018 Jun 26 [cited 2022 Jan 14]. <https://laviedesidees.fr/Le-vaccin-a-marche-forcee.html>.

[34] OMS. Etude anthropologique sur la vaccination; 2019.

[35] OMS. Evaluation des occasions manquées de vaccination (OMV) à Madagascar. Résultats préliminaires; 2019.

